# Improving Stored Product Insect Pest Management: From Theory to Practice

**DOI:** 10.3390/insects10100332

**Published:** 2019-10-04

**Authors:** David W. Hagstrum, Christos G. Athanassiou

**Affiliations:** 1 Department of Entomology, Kansas State University, Manhattan, KS 66502, USA; 2 Laboratory of Entomology and Agricultural Zoology, Department of Agriculture, Crop Production and Rural Environment, University of Thessaly, Phytokou str., Nea Ionia, 38446 Magnissia, Greece

Integrated pest management (IPM) is being more widely used for managing stored product insects [1,2] and in some cases may be easier for stored products than for crops or orchards. However, the development of a new method, improving existing methods and basic research on insect pests and their natural enemies that facilitate better efficacy and pest management decisions have dominated research on managing stored product insect pests. A recent editorial review criticized IPM for focusing on the discovery of new pest management methods without much regard for making decisions about how to use them [3]. Insect pest management can be also improved by making better decisions about when and where pest management is needed or which control method to use. Many industry practices can influence the efficacy of pest management program for raw [4] and processed commodities [5] and must be considered when designing a pest management program. Encouraging end-users to use a new method or to use existing methods better also improves pest management [6]. A recent paper on aerosols is an example of a paper with improvements that can be immediately adopted by the aerosol applicators [7].

## 1. Designing a Monitoring Program

Since insects generally are not uniformly distributed [8,9], random sampling can provide an unbiased sample of an insect population, but these samples may not be representative of the insect population. Information on insect distribution can be used to develop a more representative stratified random sampling plan. Although insect distribution has been described for various types of storage facilities, recommendations for localized treatments often are not provided.

Accurate estimation of insect density often requires more sampling than is feasible and a practical alternative is determining whether a pest population will soon reach the density at which economic losses occur. When the population is close to an economic threshold (ET), many samples are required to determine whether density is above or below ET and sampling again later is generally recommended, but when density is close to ET, control might be just as appropriate. ET has been determined for eight insect species that infest raw commodity storages, processing plants, or retail businesses [2,10]. The thresholds for traps were higher than were those for commodity samples and lower for processed commodities than for raw commodities. ETs are used by most rice storage facilities in California [11], and there are several ET levels that are internally determined by different durable product industries and regulatory agencies. The Carvalho et al. paper showed that radiographic analysis is efficient in the detection of damage caused by the most important insect pest species for stored maize globally, the maize weevil, *Sitophilus zeamais* Motschulsky. 

Traps catch can be influenced by many factors which need to be considered in interpreting the trap catch and making pest management decisions [12]. Automation has been considered as a means of developing more cost-effective insect monitoring programs [13,14,15]. Njoroge et al.’s paper demonstrated that the use of acoustic technology with *S. zeamais* and the larger grain borer, *Prostephanus truncatus* (Horn) and visual surveys and pitfall traps with other insect pest species can help managers identify and target infestations within their warehouses, enabling them to reduce postharvest losses.

## 2. Choosing Pest Management Method

Understanding the primary advantages of each insect pest management method can make choosing the best pest management method easier [16]. Residual insecticides and insect-resistant packaging provide long-term protection. Natural enemies, fumigation, and heat penetrate areas where insects hide. By eliminating hiding places of insects and reducing insect densities, sanitation and pest exclusion can increase the effectiveness of additional methods that are to be applied. Aeration of grain stored in bins and warehouses, ionizing radiation of stored commodities including grain, spices, and nuts; heat treatments and impact and pest removal used in food-processing facilities can be more expensive than other pest management methods requiring long-term planning and expensive equipment purchases in addition to operating costs.

Population growth can be forecasted using the effects of temperature and commodity moisture content on insect life history data. Life history has been studied for 248 species [17]. Studies also have evaluated the suitability of diets for the development of 92 pest species [18]. Computer simulation models predicting population growth have been developed for 22 pests and natural enemy species [19,20,21,22,23,24,25,26,27] and could be developed for many more insect species, putting this basic research on life histories to work for pest management. Life history data can also be used to estimate when a commodity was infested [28]. Aeration, residual insecticides, and fumigation have been simulated for 5 pest species [19]. Using computer simulation, the effectiveness of different pest management methods can be compared before deciding which of them to use. For the Indianmeal moth, *Plodia interpunctella* (Hübner) populations, the influence of diet on population growth [29], and the timing and frequency of insecticide treatments on their effectiveness have been simulated [30]. The cost-effectiveness of various alternatives to methyl bromide fumigation have been compared using computer simulations [31]. The profitability of the pest management strategies used at commercial grain elevators have been compared using these simulation models [32].

Several papers in this special issue and the previous special issue in 2016 on shift to nonchemical control of stored product insects [33] provide information useful in choosing a pest management method and deciding how to use it. Low temperatures are a primary nonchemical pest control method so lower temperature thresholds are relevant to pest control as well as monitoring and pest risk modeling. A paper by Stejskal et al. summarizes the lower temperature thresholds for 121 species of stored product and food industry pests. Lowest thresholds were those for Acari (6.8 °C) and Diptera (8.1 °C), followed by Lepidoptera (11.3 °C) and Psocoptera (13.8 °C), and the highest were those for Coleoptera (14 °C) and Blattodea (15 °C). Temperature thresholds were lowest for walking and greater for trap capture of walking insects followed by development < population growth < flight < capture of flying pests. Hermetic technologies are being promoted in Africa as safer and effective methods for grain storage on smallholder farms. A paper by Mutambuki et al. found that Purdue Improved Crop Storage (PICS) bags were superior to treated and untreated controls of polypropylene and jute bags in suppressing *P. truncatus* and *S. zeamais* development, maize grain damage and weight loss during storage. Inert dusts have replaced residual insecticides in situations where insects have become resistant to insecticides, or where conventional insecticides cannot be used, such as in the case of organic products. López-García et al.’s paper scales up data on the effects of nanoparticle-based inert dust on rice weevil, *Sitophilus oryzae* (L.) from a petri dish to 400 g of wheat in laboratory bioassays.

The paper by Savković et al. showed that the bean weevil, *Acanthoscelides obtectus* (Say) can sustain population growth on two suboptimal plant hosts (chickpeas and mung beans). Populations grew more readily on chickpea than on mung bean and insects from mung bean did better when given common bean host again. The ability to overcome the chemical defenses of a new host may be an important consideration in developing a pest management program. The paper by Rodríguez-González et al. finds that essential oils from basil and citronella killed 70% of adults in two weeks, reduced damage and may be useful as part of the integrated management of *A. obtectus* to reduce post-harvest losses in common bean. Romani et al. found that essential oils from two plant species in the genus *Clinopodium* showed a strong repellent effect towards adult *S. zeamais*, with a clear involvement of the insect antennal olfactory system. Such studies will encourage further registration and use of natural resource-based active ingredients as components of an improved IPM in storage and processing facilities. Although their use in developed counties is negligible for stored product protection, plant extracts are widely used in many parts of the developing world, in many types of facilities and commodities.

The paper by Mbata and Warsi reviews factors that need to be considered for the use of the parasitoids *Habrobracon hebetor* (Say) and *Pteromalus cerealellae* (Ashmead) in a biological control-based pest management program, which is particularly important for organic food production. The paper by Hasan et al. shows how irradiation of moth larval hosts might improve a parasitoid rearing program in the case of *H. hebetor*.

The khapra beetle, *Trogoderma granarium* Everts, is a quarantine pest. The Arthur et al. paper showed that 2 new chemical treatments can be utilized in management programs when *T. granarium* infestations are detected. With related species, a paper by Lanka et al. found that pyrethrin + methroprene aerosol was more effective on the warehouse beetle, *Trogoderma variabile* Ballion than the larger cabinet beetle, *Trogoderma inclusum* (LeConte). Differences in effectiveness and residual persistence with particle size highlight the need for attaining optimal particle size to improve overall efficacy of aerosol mixtures containing methoprene.

## 3. Choosing a Combination of Pest Management Methods

Research on synergistic combinations of pest management methods has been expanded recently [34]. We found only 9 studies published prior to 1971, 12 from 1971–1980, 18 from 1981–1990, 39 from 1991–2000, 74 from 2001–2010 and 54 since 2010. A dozen examples of combinations are given in Table 7.12 [16]. Computer simulation models can be used to compare different combinations. Papers in this special issue provide information useful in choosing a combination of pest management methods. The paper by Arthur was used by the registrant in the United States to modify the commercial formulation of combinations of methoprene and deltamethrin as long-term protectants of wheat, corn, and rice. The paper by Morrison et al. reviews the literature on the effect of sanitation on other pest management practices, finds that the effectiveness of other methods is generally reduced by poor sanitation and concludes that sanitation should be of the utmost importance for food facility managers. The Pierattini et al. paper shows essential oils increase the effectiveness of diatomaceous earth against the granary weevil, *Sitophilus granarius* (L.). Francikowski et al.’s paper showed that essential oil mixtures were generally more repellent than single oil, with lemon and vanilla 1:1 mixture acting as the strongest repellent. Data on the effects of essential oils on locomotor activity may lead to better design of pull-push strategies in stored-product pest management, a method for which there are very few data available.

## 4. Timing Pest Management

Computer simulation models can be used to optimize the timing of pest management. Computer simulations showed that a one-month later harvest (because of differences in latitude) or a one-month delay in fumigation can result in stored-product insect populations that are roughly 5- to 25-fold smaller [35]. Delayed harvest or fumigation reduces the time available for insect populations in the stored grain to grow before the grain can be cooled by aeration or winter temperature. Populations are 5- to 25-fold larger for every month that cooling by aeration is delayed. Simulations showed that releasing the parasitoid *Cephalonomia waterstoni* Gahan on day 20 reduced the rusty grain beetle, *Cryptolestes ferrugineus* (Stephens) population by 75% more than releasing the same number of parasitoids on day 40 [36]. Releasing parasitoids when the host first reaches a susceptible stage prevents most of the pests from reproducing.

## 5. Targeting Pest Management

Population monitoring often indicates that an insect pest is not a problem throughout a facility and that pest management should be focused on certain areas. The software Stored Grain Advisor Pro correctly predicted that only 47 of the 399 elevator bins in Kansas and 45 of the 133 bins in Oklahoma needed to be fumigated [37]. In a pet food mill, insect pests were most abundant in two areas, continuously in the production area and sporadically in the warehouse, both related to the presence of refuges and food resources [38], as a result of poor sanitation. The majority (81%) of male almond moth, *Cadra cautella* (Walker) were captured in only 3 of the 35 rooms of a confectionary factory [39]. Increased moth captures were associated with birdseed and chicken feed in grocery distribution warehouses [40]. Variation in beetle captures among trap locations in a food warehouse was probably due to the movement of food products into and out of the warehouse, and movement of products and beetle populations within the warehouse [41]. Stored product insects were concentrated in pet food areas of grocery stores [42]. In the surveyed department stores, infestations generally were associated with food products in or near the pet department and in the pet stores, infestations were generally associated with cat food, dog food, and horse feed [43]. Jian’s paper shows that insect movement inside and outside bulks of grain and processed products influences pest management decisions. Rosi et al. found that nearly all adult *Carpophilus hemipterus* (L.) contaminating harvested dates can be eliminated by radiofrequency heating causing them to emigrate from the dates. In the Gerken and Campbell paper, data collected over a 10-year period at a flour mill show that both *P. interpunctella* and *T. variabile*, inside and outside the mill, are highly influenced by season, with peaks of insect captures during the warm season (April through September) that can be targeted for pest management. Populations of these insects may be moving in and out of the structures so that fumigation impacts only a small portion of the overall population and tactics targeting immigration may be an important addition to a pest management program.

Information on the distribution of an insecticide or fumigant also can be useful in making pest management decisions [7,44]. Arthur’s paper showed that progeny production and feeding damage by the lesser grain borer, *Rhyzopertha dominica* (F.) and the Angoumois grain moth, *Sitotroga cerealella* (Olivier) decreased as the proportion of rice treated with deltamethrin increased. Results show that coverage is important and that the new deltamethrin formulation could be used for protection of brown rice. However, *S. cerealella* may be less susceptible to deltamethrin compared to *R. dominica*. The paper by Scheff et al. demonstrates a practical use of aerosol insecticides and their potential as effective alternatives to structural fumigations for adult confused flour beetle, *Tribolium confusum* Jacquelin duVal, in a commercial rice mill. Records of spatial variation in spray concentration showed that strategies to improve the consistency of coverage could improve effectiveness.

Insect pests are often managed by phosphine fumigation and phosphine levels must be monitored to protect workers and achieve maximum insect control. A survey of 28 grain elevator managers in Oklahoma indicated that electronic-type phosphine monitoring devices were more convenient than the tube-type monitoring device and that workers at these elevators felt that electronic-type were better at ensuring worker safety [45]. An economic analysis showed that the cost of electronic-type monitoring devices was generally less than the tube-type monitoring device because the costs of the Mine Safety Appliances (MSA) tube quickly exceeded the purchase price and maintenance costs of the electronic-type monitoring devices. The paper by Brabec et al. demonstrated that a new automated monitoring system using electronic-type phosphine monitoring device was as reliable as the traditional manual tube-type monitoring. Automated monitoring may improve pest management by providing a more detailed picture than manual monitoring of the spatial variation in phosphine concentrations.

Early detection can be important in managing insecticide resistance and a paper by Cato et al. developed a quick test for resistance based on the time for *T. castaneum* adults to become immobile when exposed to a high concentration of phosphine. Times for the three strong resistant populations were all over 100 min, compared to 60 min or less for susceptible and weak resistant populations. This is one of the few rapid evaluation tests for phosphine resistance that differentiates strong from weak resistance.

## 6. Improving Pest Management

Relatively few studies are focused on optimizing pest management decisions for each facility because most research is focused on insect behavior, biology, and ecology or the development of pest monitoring and management methods. This editorial examines how the papers in this Special Issue can be important in tailor-making pest management decisions for each storage, transport, and processing facility. Stored product insect management starts with monitoring for the presence of insects in a facility and their resistance to any insecticide that may be applied. Monitoring should be done right before and after pest management to demonstrate efficacy.

## References

[1] Athanassiou C.G., Arthur F.H. (2017). Recent Advances in Stored Product Protection.

[2] Hagstrum D.W., Phillips T.W. (2017). Evolution of Stored-Product Entomology: Protecting the World Food Supply. Annu. Rev. Entomol..

[3] Peterson R.K.D., Higley L.G., Pedigo L.P. (2018). Whatever Happened to IPM?. Am. Entomol..

[4] Hagstrum D.W., Reed C., Kenkel P. (1999). Management of stored wheat insect pests in the USA. Integr. Pest Manag. Rev..

[5] Trematerra P., Fleurat-Lessard F. (2015). Food industry practices affecting pest management. Stewart Postharvest Rev..

[6] Hagstrum D.W., Subramanyam B.H., Hagstrum D.W., Phillips T.W., Cuperus G. (2012). Role of extension educators and consultants. Stored Product Protection.

[7] Campbell J.F., Arthur F.H., Brabec D., Scheff D., Adler C.S., Opit G., Fürstenau B., Müller-Blenkle C., Kern P., Arthur F.H., Athanassiou C.G., Bartosik R., Campbell J., Carvalho M.O. Temporal and Spatial Patterns in Aerosol Insecticide Droplet Distribution: Modifying Application Strategies to Improve Coverage and Efficacy. Proceedings of the 12th International Working Conference on Stored Product Protection (IWCSPP), Berlin, Germany, 7–11 October 2018.

[8] Athanassiou C.G., Kavallieratos N.G., Sciarretta A., Palyvos N.E., Trematerra P. (2011). Spatial Associations of Insects and Mites in Stored Wheat. J. Econ. Entomol..

[9] Hagstrum D.W., Milliken G.A., Wadell M.S. (1985). Insect distribution in bulk stored wheat in relation to detection or estimation of abundance. Environ. Entomol..

[10] Hagstrum D.W., Flinn P.W. (2014). Modern stored-product insect pest management. J. Plant Prot. Res..

[11] Espino L., Greer C.A., Mutters R., Thompson J.F. (2014). Survey of rice storage facilities identifies research and education needs. Calif. Agric..

[12] Storedpoductinsects.com: Interpretation of trap catch. https://storedproductinsects.com/biology/interpretation-of-trap-catch/.

[13] Flinn P.W., Opit G.P., Throne J.E., Lorini I., Bacaltchuk B., Beckel H., Deckers D., Sundfeld E., dos Santos J.P., Biagi J.D., Celaro J.C., Faroni L.R.D.A., Bortolini L.d.O.F. (2006). Integrating the Stored Grain Advisor Pro expert system with an automated electronic grain probe trapping system. Proceedings of the 9th International Working Conference on Stored Product Protection in Campinas, Sao Paulo, Brazil, 15–18 October 2006.

[14] Eliopoulos P.A., Potamitis I., Rigakis I., Adler C.S., Opit G., Fürstenau B., Müller-Blenkle C., Kern P., Arthur F.H., Athanassiou C.G., Bartosik R., Campbell J., Carvalho M.O. Automated detection and monitoring of grain beetles using a “smart” pitfall trap. Proceedings of the 12th International Working Conference on Stored Product Protection (IWCSPP), Berlin, Germany, 7–11 October 2018.

[15] Hagstrum D.W., Flinn P.W., Shuman D. (1996). Automated monitoring using acoustical sensors for insects in farm-stored wheat. J. Econ. Entomol..

[16] Hagstrum D.W., Klejdysz T.Z., Subramanyam Bh Nawrot J. (2013). Atlas of Stored-Product Insects and Mites.

[17] Storedpoductinsects.com: Life histories of stored product insects and mites. https://storedproductinsects.com/biology/life-histories-of-stored-product-insects-and-mites/.

[18] Storedpoductinsects.com: Suitability of food. https://storedproductinsects.com/new-species/suitability-of-foods/.

[19] Hagstrum D.W., Subramanyam B.H. (2006). Fundamentals of Stored Product Entomology.

[20] Flinn P., Phillips T., Hagstrum D., Arthur F., Throne J., Donahaye E.J., Navarro S., Leesch J. (2001). Modeling the effects of insect stage and grain temperature on phosphine-induced mortality for Rhyzopertha dominica. Proceedings of the International Conference Controlled Atmosphere and Fumigation in Stored Products, Fresno, CA, USA, 29 October–3 November 2000.

[21] Flinn P.W., Campbell J.F., Throne J.E., Subramanyam B., Carvalho M.O., Fields P.G., Adler C.S., Arthur F.H., Athanassiou C.G., Campbell J.F., Fleurat-Lessard F., Flinn P.W., Hodges R.J., Isikber A.A. (2010). Simulation model of the red flour beetle in flour mills. Proceedings of the 10th International Working Conference on Stored Product Protection, Estoril, Portugal, 27 June–2 July 2010.

[22] Kuzmanov D., Dimitrov N. (2009). Forecasting the necessity of grain fumigation during storage. Czech J. Food Sci..

[23] Limonta L., Locatelli D., Broglia T., Baumgartner J. (2009). Cohort development models for integrated *Corcyra cephalonica* population management (Stainton). Boll. Zool. Agrar. Bachic..

[24] Throne J.E., Arbogast R.T. (2010). A Computer model for simulating population development of the Indianmeal Moth (Lepidoptera: Pyralidae) in Stored Corn. J. Econ. Entomol..

[25] Thorne J., Fulford G., Ridley A., Schlipalius D., Collins P., Carvalho M.O., Fields P.G., Adler C.S., Arthur F.H., Athanassiou C.G., Campbell J.F., Fleurat-Lessard F., Flinn P.W., Hodges R.J., Isikber A.A. (2010). Life stage and resistanceeffects in modelling phosphine fumigation of Rhyzopertha dominica (F.). Proceedings of the 10th International Working Conference on Stored Product Protection, Estoril, Portugal, 27 June–2 July 2010.

[26] Pekar S., Zd’arkova E. (2004). A model of the biological control of *Acarus siro* by *Cheyletus eruditus* (Acari: Acaridae, Cheyletidae) on grain. J. Pest Sci..

[27] Shi M., Collins P.J., Ridsdill-Smith J. (2012). Individual-based modelling of theefficacy of fumigation tactics to control lesser grain borer (*Rhyzopertha dominica*) in stored grain. J. Stored Prod. Res..

[28] Defilippo F., Pinna M., Calzolari M., Dottori M., Bonilauri P., Vanin S., Bourguignon L., Charabidze D., Whitaker A., Aubernon C., De Carvalho Moretti T., Pasquerault T., Amendt J., Brown K., Campobasso C. (2017). Stored-product forensic entomology: Study of development of Plodia interpunctella (Lepidoptera: Pyralidae) and use of data in determining time intervals of food infestation. Proceedings of the 14th Meeting European Association for Forensic Entomology, Treviso, Italy, 7–10 June 2017.

[29] Fontenot E.A., Arthur F.H., Nechols J.R., Throne J.E. (2012). Using a population growth model to simulate response of *Plodia interpunctella* Hübner to temperature and diet. J. Pest Sci..

[30] Fontenot E.A., Arthur F.H., Nechols J.R., Throne J.E. (2012). Using a population growth model to simulate response of *Plodia interpunctella* Hübner populations to timing and frequency of insecticide treatment. J. Pest Sci..

[31] Niu L. (2016). Alternatives to Methyl Bromide Fumigation for Insect Control in Rice and Wheat Processing Facilities: An Economic Optimization. Master’s Thesis.

[32] Kenkel P., Adam B.D., Cuperus G., Fargo W.S. (1991). A Risk Analysis of Insect Control Strategies in Stored Wheat.

[33] Palmeri V., Athanassiou C.G., Kavallieratos N.G., Campolo O. (2016). Alternatives to Chemical Control of Stored-Product Insects, Insects. https://www.mdpi.com/journal/insects/special_issues/stored_product.

[34] Storedproductinsects. https://storedproductinsects.com/new-species/combining-pest-control-methods/.

[35] Hagstrum D.W., Flinn P.W. (1990). Simulations comparing insect species differences in response to wheat storage conditions and management practices. J. Econ. Entomol..

[36] Flinn P.W., Hagstrum D.W. (1995). Simulation model of *Cephalonomia waterstoni* (Hymenoptera: Bethylidae) parasitizing the rusty grain beetle (Coleoptera: Cucujidae). Environ. Entomol..

[37] Flinn P.W., Hagstrum D.W., Reed C., Phillips T.W. (2007). Stored Grain Advisor Pro: Decision support system for insect management in commercial grain elevators. J. Stored Prod. Res..

[38] Belda C., Ribes-Dasi M., Riudavets J. (2011). Improving pest management in pet food mills using accurate monitoring and spatial analysis. J. Stored Prod. Res..

[39] Bowditch T.G., Madden J.L. (1996). Spatial and temporal distribution of *Ephestia cautella* (Walker) (Lepidoptera: Pyralidae) in a confectionary factory: Causal factors and management implications. J. Stored Prod. Res..

[40] Vick K., Koehler P., Neal J. (1986). Incidence of stored-product Phycitinae moths in food distribution warehouses as determined by sex pheromone-baited traps. J. Econ. Entomol..

[41] Arthur F.H., Campbell J.F., Toews M.D. (2014). Distribution, abundance, and seasonal patterns of stored product beetles in a commercial food storage facility. J. Stored Prod. Res..

[42] Platt R.R., Cuperus G.W., Payton M.E., Bonjour E.L., Pinkston K.N. (1998). Integrated pest management perceptions and practices and insect populations in grocery stores in South-central United States. J. Stored Prod. Res..

[43] Arbogast T.A., Kendra P.E., Mankin R.W., McGovern J.E. (2000). Monitoring insect pests in retail stores by trapping and spatial analysis. J. Econ. Entomol..

[44] Kaloudis E., Bantas S., Athanassiou C.G., Agrafioti P., Sotiroudas V., Adler C.S., Opit G., Furstenau B., Muller-Blenkle C., Kern P., Arthur F.H., Athanassiou C.G., Bartosik R., Campbell J., Carvalho M.O. Modeling the distribution of phosphine in cylindrical grain silos with CFD methods for precision fumigation. Proceedings of the 12th International Working Conference for Stored Product Protection, Berlin, Germany, 7–11 October 2018.

[45] Danley R. (2002). Economic Evaluation of Phosphine Fumigant Monitoring Devices Used in Oklahoma Commercial Grain Elevators. Master’s Thesis.

